# Konsensus-Statement der Österreichischen Gesellschaft für Rheumatologie und Rehabilitation zum Management des erhöhten kardiovaskulären Risikos bei rheumatoider Arthritis, Psoriasisarthritis und Spondyloarthritis

**DOI:** 10.1007/s00508-026-02718-8

**Published:** 2026-04-02

**Authors:** Boris Lindner, Mathias Ausserwinkler, Christina Siess, Kai Ammerer, Monika Esposito, Josef Hermann, Rainer Hintenberger, Matthias Komposch, Lukas Lanser, Helga Lechner-Radner, Caroline Peter, Gersina Rega-Kaun, David Reinhart-Mikocki, Simon Schedl, Michaela Stögerer-Lanzenberger, Jens Thiel, Alexander Niessner

**Affiliations:** 1 Zentrum für Diagnostik und Therapie rheumatischer Erkrankungen, Wiener Gesundheitsverbund, Klinik Hietzing, Wien, Österreich; 2 Abteilung für Innere Medizin, Elisabethinen-Krankenhaus Klagenfurt, Klagenfurt, Österreich; 3 Abteilung für Innere Medizin I, Paracelsus Medizinische Universität Salzburg, Salzburg, Österreich; 4 Interne III-Abteilung für Nephrologie und Rheumatologie, Ordensklinikum Linz Standort Elisabethinen, Linz, Österreich; 5 Klinische Abteilung für Rheumatologie und Immunologie, Medizinische Universität Graz, Graz, Österreich; 6 1. Medizinische, Abteilung, Hanusch-Krankenhaus, Medizinische Fakultät, Johannes Kepler Universität, Linz, Österreich; 7 Universitätsklinik für Innere Medizin II, Medizinische Universität Innsbruck, Innsbruck, Österreich; 8 Universitätsklinik für Innere Medizin III, Abteilung für Rheumatologie, Medizinische Universität Wien, Wien, Österreich; 9 5. Medizinische Abteilung mit Endokrinologie, Rheumatologie und Akutgeriatrie, Wiener Gesundheitsverbund, Klinik Ottakring, Wien, Österreich; 10 3. Medizinische Abteilung, Innere Medizin mit Stoffwechselerkrankungen, Endokrinologie und Nephrologie, Wiener Gesundheitsverbund, Krankenhaus Hietzing, Wien, Österreich; 11 Rheumatologie und Klinische Immunologie, Universitätsklinikum Freiburg, Freiburg, Deutschland; 12 Abteilung für Kardiologie, Universitätsklinik für Innere Medizin II, Medizinische Universität Wien, Wien, Österreich; 13 2. Medizinische Abteilung mit Kardiologie und Intensivmedizin, Wiener Gesundheitsverbund, Klinik Landstrasse, Wien, Österreich

**Keywords:** Herzinfarktrisiko bei rheumatoider Arthritis, Empfehlungen kardiovaskuläres Risiko, Herzinsuffizienz bei rheumatischen Erkrankungen, Vorhofflimmern bei rheumatischen Erkrankungen, Lipideinstellung bei rheumatischen Erkrankungen, Risk of myocardial infarction in rheumatoid arthritis, Recommendations cardiovascular risk, Heart failure in rheumatic diseases, Atrial fibrillation in rheumatic diseases, Treatment of dislipidemia in rheumatic diseases

## Abstract

**Hintergrund:**

Chronisch rheumatische Erkrankungen sind mit einem erhöhten Risiko für kardiovaskuläre Erkrankungen assoziiert, die signifikant zu einer erhöhten Morbidität und Mortalität beitragen. Obwohl internationale Empfehlungen bereits existieren, fehlen konkrete Handlungsanleitungen für die klinische Praxis, auch aufgrund heterogener Evidenz und noch nicht vollständig geklärter Pathomechanismen der kardiovaskulären Ereignisse.

**Ziel:**

Das Ziel war die Erstellung eines Expertenkonsensus, um praxisnahe Empfehlungen zur Prävention, Screening und zum Management des erhöhten kardiovaskulären Risikos bei rheumatoider Arthritis, Psoriasisarthritis und Spondyloarthritis zu formulieren.

**Methoden:**

Zwischen 2023 und 2025 arbeiteten 21 Expert*innen aus verschiedenen Fachrichtungen (Rheumatologie, Kardiologie, Radiologie, Nephrologie, Endokrinologie) im Rahmen des Arbeitskreises „Rheuma und Herz“ der Österreichischen Gesellschaft für Rheumatologie und Rehabilitation. Ein strukturierter Workflow wurde in mehreren Meetings entwickelt und ein Delphi-Prozess gestartet. Fünfzig Statements wurden auf einer Likert-Skala (10 Punkte) zur Abstimmung gebracht; schließlich haben nach einer Überarbeitung alle Statements eine Zustimmung von > 90 % erreicht. Nach Abschluss des Prozesses wurde der Workflow in ein Konsensuspapier überführt.

**Ergebnisse:**

Das Gremium schlägt ein dreiphasiges Modell der kardiovaskulären Versorgung vor. Phase 1 (Erstdiagnose einer rheumatischen Erkrankung) legt den Schwerpunkt auf das rasche Erreichen einer Remission, die Berücksichtigung kardiovaskulärer Effekte antirheumatischer Therapien sowie eine frühzeitige Lebensstilberatung. Die kardiovaskulären Profile antirheumatischer Medikamente werden in einer Heat-Map zusammengefasst. Phase 2 (rheumatische Erkrankung mit niedriger Krankheitsaktivität oder in Remission) konzentriert sich auf ein systematisches kardiovaskuläres Risikoscreening ab einem Alter von ≥ 40 Jahren unter Verwendung von SCORE2 oder SCORE2-OP mit einem krankheitsspezifischen Multiplikationsfaktor von 1,5 für RA, PsA und bei radiographischer axialer SpA. Die Patientinnen und Patienten werden gemäß den von der ESC definierten Risikokategorien stratifiziert, mit definierten Upgrade-Kriterien. Phase 3 (erweiterte Diagnostik und Primärprävention) verknüpft die Risikokategorien mit LDL-Cholesterin-Zielwerten sowie einer angepassten Diagnostik mit dem Ziel der frühzeitigen Erkennung von Atherosklerose, Arrhythmien und Herzinsuffizienz. Bei niedrigem Atheroskleroserisiko sollte eine Blutdruckmessung erfolgen, bei moderatem Risiko zusätzlich eine Karotissonographie und bei hohem oder sehr hohem Risiko zusätzlich ein EKG. Zum Screening auf Herzinsuffizienz wird eine liberale Bestimmung von NT-proBNP empfohlen; bei erhöhten Werten oder spezifischen Fragestellungen sollte eine gezielte Echokardiographie durchgeführt werden. Ein opportunistisches Screening mittels Pulspalpation wird ebenfalls für Patientinnen und Patienten mit erhöhtem Risiko für Vorhofflimmern empfohlen. Das EKG oder elektronische Devices können Hinweise auf Arrhythmien und Erregungsleitungsstörungen liefern.

**Fazit:**

Dieser Konsensus bietet einen strukturierten, krankheitsspezifisch angepassten und pragmatischen Ansatz für das Management des kardiovaskulären Risikos bei RA, PsA und SpA. Durch die Integration der Kontrolle der Grunderkrankung, der Risikostratifizierung und einer maßgeschneiderten Diagnostik zielt er darauf ab, die frühzeitige Erkennung und Prävention kardiovaskulärer Komorbiditäten in der rheumatologischen Praxis zu verbessern.

## Abstract (en)

**Background:**

Chronic inflammatory rheumatic diseases are associated with an increased risk of cardiovascular diseases (CVD), which significantly contribute to an increased morbidity and mortality. Although international recommendations exist, disease-specific and pragmatic guidance for the routine clinical practice are lacking due to heterogeneous evidence and incompletely understood pathophysiological mechanisms of cardiovascular events.

**Objective:**

The aim was to develop an expert consensus providing practical recommendations for the prevention, screening and management of the increased cardiovascular risk in patients with rheumatoid arthritis (RA), psoriatic arthritis (PsA) and spondyloarthritis (SpA).

**Methods:**

Between 2023 and 2025 a multidisciplinary panel of 21 experts (rheumatology, cardiology, radiology, nephrology and endocrinology) convened under the Working Group “Rheumatism and Heart” of the Austrian Society for Rheumatology and Rehabilitation. A structured workflow was developed through iterative meetings and refined using a Delphi process. A total of 50 statements were rated on a 10-point Likert scale; all achieved > 90% level of agreement after revision. The final consensus was consolidated into a structured clinical workflow.

**Results:**

The panel proposes a three-phase cardiovascular care model. Phase 1 (initial diagnosis of a rheumatic disease) emphasizes rapid achievement of remission, consideration of cardiovascular effects of antirheumatic treatment and early lifestyle counselling. The cardiovascular profiles of antirheumatic drugs are summarized in a heat map. Phase 2 (rheumatic disease with low disease activity or in remission) focuses on systematic cardiovascular risk screening from age ≥ 40 years using SCORE2 or SCORE2-OP, with a disease-specific multiplication factor of 1.5 for RA, PsA and radiographic axial SpA. Patients are stratified into risk categories defined by the European Society of Cardiology (ESC), with defined upgrade criteria. Phase 3 (advanced diagnostics and primary prevention) links risk categories to low-density lipoprotein (LDL)-cholesterol targets and tailored diagnostics aiming for early detection of atherosclerosis, arrhythmias and heart failure. In cases of a low risk for atherosclerosis blood pressure monitoring should be performed, at moderate risk additional carotid ultrasound and at high or very high risk an additional electrocardiography (ECG). To screen for heart failure a liberal amino-terminal pro-brain natriuretic peptide (NT-proBNP) measurement is recommended and in cases of elevated results or specific questions selective echocardiography is recommended. Opportunistic screening through pulse palpation is also recommended for patients at increased risk of atrial fibrillation. The ECG or electronic devices can provide evidence of arrhythmias and conduction abnormalities.

**Conclusion:**

This consensus provides a structured, disease-specific and pragmatic approach to cardiovascular risk management in RA, PsA and SpA. By integrating inflammation control of the underlying disease, risk stratification and tailored diagnostics, it aims to improve early detection and prevention of cardiovascular comorbidities in routine rheumatological practice.

## Einleitung

Rheumatische Erkrankungen beeinträchtigen das Leben von Patient*innen nicht nur durch ihre primären Manifestationen, sondern auch durch ein erhöhtes kardiovaskuläres („cardiovascular disease“ [CVD]) Risiko. Zahlreiche Studien belegen, dass nahezu alle chronisch entzündlichen Erkrankungen mit einem deutlich erhöhten Risiko für kardiovaskuläre Ereignisse verbunden sind [1–5].

Im vergangenen Jahrzehnt rückten kardiovaskuläre Komorbiditäten – wie Myokardinfarkte oder Schlaganfälle – im Zusammenhang mit entzündlichen rheumatischen Erkrankungen zunehmend in den Fokus. Dies zeigt sich unter anderem in den Empfehlungen der Europäischen Gesellschaft für Rheumatologie (EULAR) [6].

Der Arbeitskreis *Rheuma und Herz* der Österreichischen Gesellschaft für Rheumatologie und Rehabilitation (ÖGR) hat einen Expertenkonsensus erarbeitet, der aktuelle Erkenntnisse und Empfehlungen zur Prävention und Behandlung des kardiovaskulären Risikos bei rheumatischen Erkrankungen zusammenfasst. Ziel dieses Konsensus ist es, das Bewusstsein für dieses Thema zu schärfen und die Versorgung der Patient*innen zu verbessern, indem praxisnahe und pragmatische Empfehlungen für den klinischen Alltag formuliert werden – auch über bestehende evidenzbasierte Leitlinien hinaus.

Aufgrund der heterogenen Datenlage und der bislang nicht vollständig geklärten Pathomechanismen kardiovaskulärer Ereignisse bei rheumatischen Erkrankungen wurde zunächst auf generelle Empfehlungen für alle entzündlich rheumatischen Erkrankungen verzichtet. Stattdessen wurde ein Konsensus für die in der Praxis häufigsten und klinisch relevantesten rheumatologischen Erkrankungen – rheumatoide Arthritis (RA), Psoriasisarthritis (PsA) und Spondyloarthritis (SpA) – erarbeitet.

Der vorliegende Konsensus legt den Schwerpunkt auf die häufig auftretende Atherosklerose, berücksichtigt jedoch auch das erhöhte Risiko für Herzinsuffizienz und Herzrhythmusstörungen [7, 8].

## Kardiovaskuläres Risiko bei rheumatischen Erkrankungen

Die Evidenz für das erhöhte kardiovaskuläre Risiko im Rahmen entzündlich rheumatischer Erkrankungen – insbesondere bei RA, PsA und SpA – ist heterogen und auch daher nicht in vollem Umfang vergleichbar, insbesondere da der Begriff „kardiovaskulär“ in Studien unterschiedlich definiert wird. Manche Arbeiten definieren neben atherosklerotischen Komorbiditäten auch Herzinsuffizienz oder Rhythmusstörungen als Endpunkte, während andere Arbeiten Surrogatmarker wie die Karotis-Intima-Media-Dicke verwenden. Trotz dieser methodischen Heterogenität zeigen nahezu alle Studien konsistent ein erhöhtes CVD-Risiko bei diesen Erkrankungen.

Die Pathogenese von kardiovaskulären Komorbiditäten ist multifaktoriell. Die systemische, oft auch subklinische Inflammation spielt eine zentrale Rolle in der Ausbildung atherosklerotischer Läsionen. Hinzu kommen metabolische Veränderungen wie Dyslipidämien, die oft aufgrund des Lipidparadoxons – einer mit der Entzündungsaktivität assoziierten Senkung der Cholesterinspiegel – häufig unterschätzt oder nicht erkannt werden. Auch der Einsatz antirheumatischer Therapien kann bidirektionale Auswirkungen auf das CVD-Risiko haben: NSAR (nichtsteroidale Antirheumatika) und Glukokortikoide können das CVD-Risiko erhöhen, während eine effektive krankheitsmodifizierende Therapie protektiv wirken kann.

### CVD-Risiko bei rheumatoider Arthritis

Je nach Studienlage und den gewählten Endpunkten (z. B. Myokardinfarkt, kardiovaskulär bedingter Tod, Schlaganfall, Vorhofflimmern, Herzinsuffizienz) ergeben sich teils abweichende Resultate. Es zeigt sich in den letzten Jahren ein Trend zu einer Abnahme der CVD-assoziierten Mortalität, während die Häufigkeit der Komorbiditäten selbst unverändert hoch bleibt. Das atherosklerotische Risiko, das hier im Vordergrund steht, ist im Vergleich zur Allgemeinbevölkerung etwa um den Faktor 1,5 erhöht [1, 6, 9].

### CVD-Risiko bei Psoriasisarthritis

Das erhöhte kardiovaskuläre Risiko bei Psoriasisarthritis ist gut belegt. Untersuchungen in rein dermatologischen Kohorten (Psoriasis vulgaris) zeigen ein erhöhtes CVD-Risiko, das bei Psoriasisarthritis zumindest in vergleichbarer Größenordnung vorzuliegen scheint. Zusätzlich finden sich bei Patient*innen mit Psoriasis oder Psoriasisarthritis häufig metabolische Begleiterkrankungen wie Adipositas, Hyperurikämie und Dyslipidämie, die ihrerseits atherosklerotisch wirksam sind. Angesichts der auch hier heterogenen Datenlage wird das CVD-Risiko in diesem Konsensus mit einem Faktor von 1,5 beziffert [1, 9].

### CVD-Risiko bei Spondyloarthritis

Das kardiovaskuläre Risiko ist auch bei der Spondyloarthritis (SpA) klar dokumentiert. Neben atherosklerotischen Veränderungen treten vermehrt Rhythmusstörungen sowie Herz- und Klappeninsuffizienzen auf. Zu berücksichtigen ist jedoch, dass Patient*innen bei der Erstdiagnose – insbesondere bei axialer SpA – häufig noch sehr jung sind, sodass klinisch manifeste atherosklerotische Veränderungen oft erst viele Jahre nach Krankheitsbeginn auftreten. Vor allem eine bei der Spondyloarthritis häufig vorkommende Diagnoseverzögerung erhöht das Risiko für kardiovaskuläre Komorbiditäten [1, 9, 10].

## Bestehende internationale Empfehlungen

Die europäische Gesellschaft für Rheumatologie (EULAR) hat bereits Empfehlungen sowie ein Update zum Management des kardiovaskulären Risikos bei rheumatischen Erkrankungen veröffentlicht [6, 11]. Diese Empfehlungen sind jedoch häufig allgemein und unspezifisch formuliert, da sie mangels belastbarer Evidenz weitgehend auf Empfehlungen für die Allgemeinbevölkerung zurückgreifen.

Die Ursachen für die begrenzte Evidenz sind vielfältig. Ein wesentlicher Faktor ist die Heterogenität der Daten: Zum einen lassen sich die unterschiedlichen rheumatischen Erkrankungen nicht unter einem einheitlichen Begriff zusammenfassen, da sie offenbar mit unterschiedlichen Ausprägungen des kardiovaskulären Risikos einhergehen. Zum anderen fehlt es in den zahlreichen Publikationen an einem standardisierten kardiovaskulären Endpunkt, sodass das Risiko letztlich nur geschätzt werden kann. Darüber hinaus existieren nur wenige Interventionsstudien zur Reduktion kardiovaskulärer Komorbiditäten bei rheumatischen Erkrankungen. So war beispielsweise „eine rheumatische Grunderkrankung“ in Zulassungsstudien für Statine ein Ausschlusskriterium.

Diese Faktoren erschweren die Erstellung evidenzbasierter Leitlinien erheblich.

## Methoden

Zwischen 2023 und 2025 erarbeiteten 21 Expert*innen aus verschiedenen Fachrichtungen einen Workflow zur Reduktion des kardiovaskulären Risikos bei RA, PsA und SpA. Beteiligt waren 18 Rheumatolog*innen (Fachärzt*innen/Ärzt*innen in Ausbildung), 3 Kardiolog*innen, eine Radiologin mit Schwerpunkt Kardioradiologie, ein Nephrologe sowie 3 Endokrinologen. Mehrere Teilnehmer*innen verfügen über Additivausbildungen in mehreren Fachgebieten.

Der Workflow entstand in mehreren Online- und Präsenztreffen. Zusätzlich wurde der Arbeitskreis „Spondyloarthritis“ der ÖGR eingebunden; in einem Online-Meeting wurden spezifische Fragen zur SpA diskutiert.

Im März 2025 begann ein Delphi-Prozess: Der erarbeitete Workflow wurde in 50 Statements zusammengefasst und den Teilnehmer*innen anonym zur Abstimmung auf einer Likert-Skala (1–10; „1“ = geringste Zustimmung, „10“ = höchste Zustimmung) vorgelegt. An der ersten Runde nahmen 16 Expert*innen teil. Jene Statements, die keine ausreichende Zustimmung erhielten (< 90 %), wurden in 2 weiteren Diskussionsrunden überarbeitet und erneut zur Abstimmung gestellt. In der zweiten Abstimmungsrunde erreichten schließlich alle Statements eine Zustimmung von über 90 %. Nicht alle Teilnehmer*innen haben bei jedem Statement abgestimmt; die Summe der Abstimmenden wurde in der Tabelle der Statements im Sektor LoA (Level of Agreement) in Klammer angeführt (Tab. 1).

Nach Abschluss des Prozesses wurde der Workflow in ein Konsensuspapier überführt.

## Ergebnisse

Die kardiovaskuläre Betreuung von an RA/PsA/SpA erkrankten Patient*innen sollte in 3 Phasen gegliedert werden. Dies soll abbilden, dass ein kontinuierlicher Prozess notwendig ist, um Komorbiditäten früh zu erkennen und um auch von Beginn an diesen entgegenzuwirken.

### Phase 1: Erstdiagnose RA/PsA/SpA

**Abb. 1:**
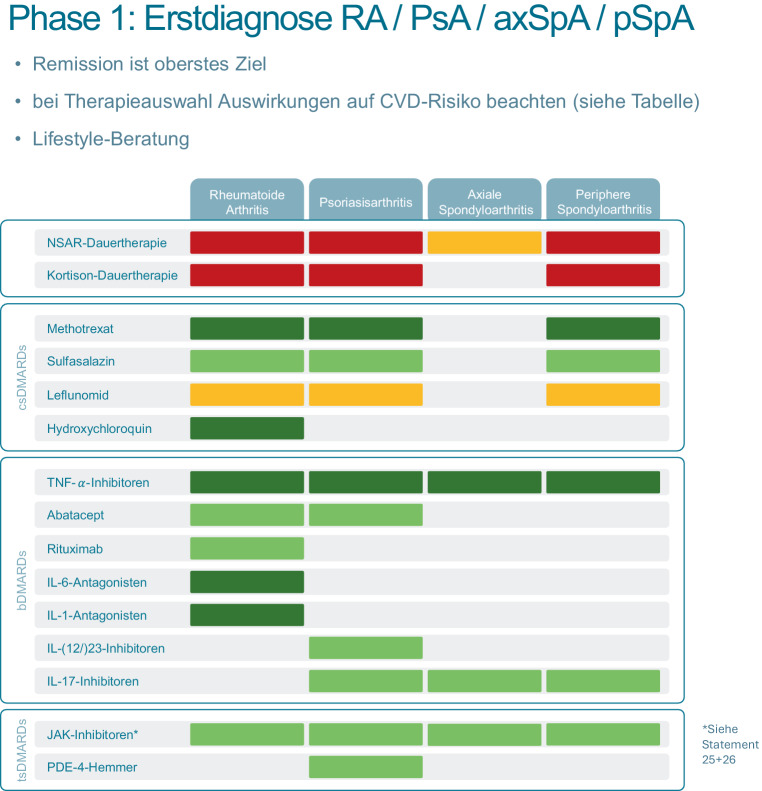
Phase 1: Zeitpunkt der Erstdiagnose rheumatoide Arthritis/Psoriasisarthritis/Spondyloarthritis

In der ersten Phase (Abb. 1), in der eine rheumatische Erkrankung neu diagnostiziert wird, werden 3 Allgemeinmaßnahmen zur CVD-Risikoreduktion empfohlen:

1. Remission sollte oberstes Ziel in der Behandlung der Grunderkrankung sein.
2. Bei der Therapiewahl sollte die kardiovaskuläre Auswirkung des antirheumatischen Medikamentes bedacht werden.
3. Eine Lebensstilberatung sollte schon zu Beginn erfolgen.

Weiters wurden die für jedes antirheumatische Medikament bekannten oder suspizierten Effekte auf das CVD-Risiko in einer Heat-Map dargestellt. Aufgrund des Einsatzes unterschiedlicher Basistherapien bei axialer und peripherer Spondyloarthritis wurde diese Erkrankung in 2 Kolumnen getrennt, auch um die (wahrscheinlich) unterschiedlichen kardiovaskulären Effekte von NSAR abzubilden.

- *Rot:* aus kardiovaskulärer Sicht ungünstig bzw. erhöht das kardiovaskuläre Risiko
- *Gelb*: aus kardiovaskulärer Sicht neutral oder unklar (divergente Daten oder fehlende Datenlage)
- Grün: aus kardiovaskulärer Sicht sicher
- *Dunkelgrün: *aus kardiovaskulärer Sicht potenziell protektiv bzw. senkt das kardiovaskuläre Risiko abseits des Effektes, der durch die Reduktion der Krankheitsaktivität zu erwarten wäre

### Phase 2: niedrige Krankheitsaktivität oder Remission

**Abb. 2:**
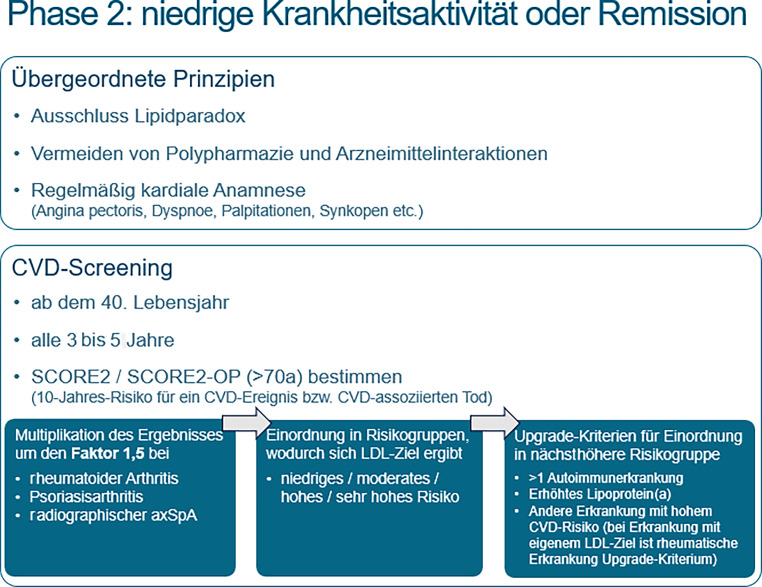
Phase 2: die rheumatische Erkrankung befindet sich in niedriger Krankheitsaktivität oder in Remission

Nach unserer Auffassung sollte ein Screening auf kardiovaskuläre Komorbiditäten erst dann erfolgen, wenn sich die Patient*innen in Remission oder zumindest in einem Zustand einer „low disease activity“ (LDA; niedrige Krankheitsaktivität) befinden (Abb. 2). Auf diese Weise lässt sich eine bessere Compliance sicherstellen, da in der frühen Phase nach Erstdiagnose (Phase 1) aufgrund der Krankheitsaktivität in der Regel noch kein ausreichendes Verständnis für Komorbiditäten erwartet werden kann.

Es wurden 3 übergeordnete Prinzipien festgelegt: Der Ausschluss eines Lipidparadoxes [12, 13], das Vermeiden von Polypharmazie [14, 15] und Medikamenteninteraktionen (wie auch die LDL(low-density-lipoprotein)-steigernde Wirkung einzelner Medikamente [16–19]) und das regelmäßige Durchführen einer kardialen Anamnese [20].

Das CVD-Screening sollte bei allen Patient*innen ab dem 40. Lebensjahr alle 3 bis 5 Jahre durchgeführt werden [6, 11, 21]. Als Instrument dient der SCORE2 (Systematic COronary Risk Evaluation) bzw. ab dem 70. Lebensjahr der SCORE2-OP (older persons) [22, 23]. Die Ergebnisse (10-Jahres-Risiko für kardiovaskuläre Erkrankungen und kardiovaskulären Tod) werden durch für die jeweilige rheumatische Erkrankung definierte Multiplikationsfaktoren [1, 6, 9] angepasst (Faktor 1,5 für RA, PsA und radiographische axiale SpA).

Anschließend erfolgt die Einordnung der Patient*innen in die von der ESC (European Society of Cardiology) definierten Risikokategorien (niedrig, moderat, hoch, sehr hoch) [24, 25]. Zusätzlich wurden sog. Upgrade-Kriterien definiert, die eine Höherstufung in eine höhere Risikokategorie ermöglichen, etwa bei Vorliegen einer zweiten Autoimmunerkrankung, anderen Erkrankungen mit hohem kardiovaskulärem Risiko oder bei erhöhtem Lipoprotein(a) [1, 26–28]. Umgekehrt können bestimmte Erkrankungen, die eigene LDL-Zielwerte erfordern (z. B. Diabetes mellitus, PAVK (periphere arterielle Verschlusskrankheit), CAVK (cerebrale arterielle Verschlusskrankheit), familiäre Hypercholesterinämie), dazu führen, dass die rheumatische Erkrankung selbst als Upgrade-Kriterium berücksichtigt wird [24].

### Phase 3: weiterführende Diagnostik und primärprophylaktische Therapie

**Abb. 3:**
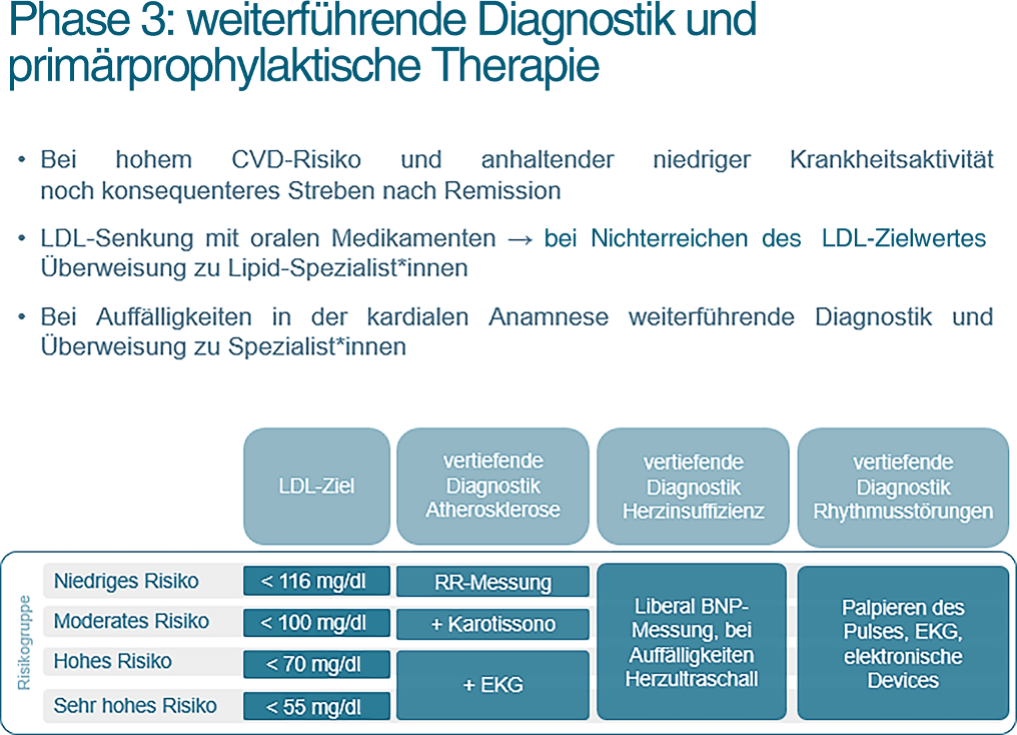
Phase 3: Empfehlungen zu weiterführender Diagnostik und primärprophylaktischer Therapie

Das durch das CVD-Screening erhaltene Resultat ergibt das 10-Jahres-Risiko für kardiovaskuläre Erkrankungen oder kardiovaskulär assoziierten Tod. Entsprechend den ESC-Leitlinien ergibt sich für die jeweiligen Risikogruppen (niedrig/moderat/hoch/sehr hoch) ein individueller Zielwert für das LDL-Cholesterin (Abb. 3).

Analog der Cholesterineinstellung sprechen wir uns auch für eine individuell angepasste weiterführende Diagnostik aus (Abb. 3). Bei niedrigem CVD-Risiko sollte der Blutdruck in regelmäßigen Abständen gemessen werden [11, 29, 30]. Bei moderatem CVD-Risiko sollte zusätzlich eine Karotissonographie vorgenommen werden [5, 31]. Bei hohem und sehr hohem CVD-Risiko sollte zusätzlich auch ein EKG (Elektrokardiogramm) durchgeführt werden [35–38].

Die Evidenz für eine weiterführende Diagnostik bei rheumatischen Erkrankungen ist gering. Weiters soll noch einmal betont werden, dass dieser Konsensus explizit kardial asymptomatische Patient*innen adressiert. Auch aus diesem Grund haben wir uns entschieden, die koronare Computertomographie bzw. den koronaren Kalziumscore, die in den ESC-Leitlinien als bildgebende Diagnostik empfohlen werden, primär für diese rheumatologische-kardiovaskuläre Screeningmaßnahme nicht zu erwähnen und Kardiolog*innen zu überlassen.

Aus unserer Sicht sollte sich die kardiovaskuläre Diagnostik nicht nur auf das Atheroskleroserisiko beschränken, sondern auch das Risiko für Rhythmusstörungen und Erkrankungen, die die Pumpfunktion des Herzens beeinträchtigen (wie eine kardiale Insuffizienz) berücksichtigt werden. Daten zum erhöhten Risiko für Herzinsuffizienz (inklusive HFpEF [„heart failure with preserved ejection fraction“]) und Vorhofflimmern bei Rheumapatient*innen sind zwar vorhanden, geben aber keinen Hinweis darauf, wann und bei wem dieses Risiko am höchsten ist. Aufgrund dieses allgemein erhöhten Risikos für Herzinsuffizienz sowie auch für Perikarditis, pulmonalarterielle Hypertension und bei SpA-Patient*innen für eine Aortenklappeninsuffizienz werden ein liberales Screening in der Risikopopulation mittels NT-proBNP (N-terminales pro-Brain Natriuretic Peptide) sowie bei erhöhten Werten in weiterer Folge eine Echokardiographie empfohlen [32–34].

Bei einem erhöhten Risiko für eine kardiale Insuffizienz könnten sonst bei Rheumapatient*innen aufgrund der Grunderkrankung und der somit eingeschränkten Mobilität Beschwerden wie Dyspnoe möglicherweise erst verzögert wahrgenommen werden.

Weiters wird ein opportunistisches Screening bei erhöhtem Risiko für Vorhofflimmern etwa durch das Palpieren des Pulses, durch Verwendung von elektronischen Devices oder durch ein EKG empfohlen. Das EKG kann zusätzliche Hinweise auf weitere Rhythmusstörungen geben.

## Statements und Abstimmungsergebnisse

**Tab. 1:** Statements und Level of Agreement (Likert-Skala, in Klammer Anzahl der Abstimmungen)

	Statement	LoA
*1*	*Remission sollte auch zur kardiovaskulären Risikoreduktion als oberstes Ziel in der Behandlung der rheumatoiden Arthritis/Psoriasisarthritis/Spondyloarthritis erachtet werden* In zahlreichen Publikationen wurde eine Assoziation zwischen der Krankheitsaktivität und dem kardiovaskulären Risiko gezeigt. So konnte nachgewiesen werden, dass sich schon vor Ausbruch einer rheumatischen Grunderkrankung gewisse Surrogatparameter für eine CVD-Erkrankung entwickeln können. Andererseits wurde gezeigt, dass bei sinkender Krankheitsaktivität weniger CVD-Ereignisse auftreten und die Mortalität sinkt. In Anbetracht der Chronizität rheumatischer Erkrankungen scheint das Erreichen einer Remission die wichtigste primärprophylaktische Maßnahme zu sein. Die Therapie anderer kardiovaskulärer Risikofaktoren soll dadurch aber keinesfalls ersetzt werden [39–42]	9,81 (16)
*2*	*Bei der Wahl der antirheumatischen Therapie sollte deren Auswirkung auf das kardiovaskuläre Risiko beachtet werden* Um schon in der ersten Phase der Betreuung einen Überblick über kardiovaskuläre Auswirkungen von antirheumatischen Medikamenten zu haben, wurde eine Heat-Map angelegt. Dafür wurden 4 Kategorien definiert *Rot:* aus kardiovaskulärer Sicht ungünstig bzw. erhöht das kardiovaskuläre Risiko *Gelb*: aus kardiovaskulärer Sicht neutral oder unklar (divergente Daten oder fehlende Datenlage) *Grün:* aus kardiovaskulärer Sicht sicher *Dunkelgrün: *aus kardiovaskulärer Sicht potenziell protektiv bzw. senkt das kardiovaskuläre Risiko abseits des Effektes, der durch die Reduktion der Krankheitsaktivität zu erwarten wäre Die Informationen zu den jeweiligen Medikamenten wurden vor allem in den Kategorien Rot/Gelb/Grün aus Sicherheitsdaten erhoben. Die Evidenz in der Kategorie „Dunkelgrün“ ist mangelhaft. Medikamente wie TNF(Tumor-Nekrose-Faktor)-Blocker weisen hierfür eine einigermaßen robuste Datenlage auf, ebenso Hydroxychloroquin. Zu Interleukin-1-Hemmern wurden Interventionsstudien, die zu kardiologischen Endpunkten durchgeführt wurden, herangezogen [43, 44]	9,75 (16)
*3*	*Schon in der Phase des Stellens einer Erstdiagnose sollte eine Lebensstilberatung angeboten werden* Nicht nur aus kardiovaskulärer, sondern auch aus rheumatologischer Sicht ist eine Lebensstilberatung schon zu Beginn einer rheumatologischen Erkrankung sinnvoll. So sollen Allgemeinmaßnahmen wie Gewichtsreduktion, Diätberatung, Nikotinstopp und vermehrte körperliche Aktivität wie Kraft- und Ausdauertraining zu einer Besserung der rheumatischen Erkrankung wie auch einer Reduktion des CVD-Risikos beitragen [45, 46]	9,5 (16)
*4*	*NSAR (insbesondere COX(Cyclooxygenase)-II-Inhibitoren) können, wenn sie bei der RA und PsA als Dauertherapie eingesetzt werden, das CVD-Risiko erhöhen, und werden als „rot“ eingestuft* Die Evidenz zur Gruppe der NSAR, insbesondere der COX-II-Inhibitoren, bezüglich des kardiovaskulären Risikos stammt größtenteils aus älteren Studien. Auf die Unterschiede zwischen einzelnen Medikamenten konnte aufgrund der Vielzahl an Substanzen nicht eingegangen werden, wohl aber auf das scheinbar höhere Risiko durch COX-II-Inhibitoren. Abgesehen von einer direkten Risikoerhöhung durch NSAR lässt aber auch der Einsatz von NSAR als Dauertherapie den Schluss zu, dass eine dauerhaft erhöhte Krankheitsaktivität der RA oder PsA vorliegen dürfte, wodurch das CVD-Risiko per se erhöht ist [47–49]	9,69 (13)
*5*	Für NSAR (insbesondere COX-II-Inhibitoren) als Dauertherapie in der Behandlung der SpA findet sich eine unklare Evidenz in der Auswirkung auf das CVD-Risiko. Insbesondere muss festgehalten werden, dass NSAR als First-Line-Therapie bei axialer SpA empfohlen werden, und bei niedrigem CVD-Risiko kein Einwand gegen eine Dauermedikation besteht; bei Patient*innen, mit einem höheren CVD-Risiko sollte der Einsatz individuell entschieden werden. Aufgrund der divergenten Datenlage werden NSAR als „gelb“ eingestuft NSAR stellten bei axialer SpA einen Grundpfeiler der medikamentösen Therapie dar; insofern muss darauf gesondert eingegangen werden. Die Datenlage zum CVD-Risiko durch NSAR bei axSpA ist divergent. Aus unserer Sicht besteht kein Einwand gegen einen Einsatz bei jungen Patient*innen, ohne CVD-Risikofaktoren, da es auch Hinweise gibt, dass vor allem bei Patient*innen mit erhöhten Entzündungsparametern NSAR das CVD-Risiko sogar senken könnten. Der Einsatz von NSAR als Dauertherapie bei axSpA-Patient*innen mit erhöhtem CVD-Risiko sollte aus unserer Sicht individuell entschieden werden [50–52]	9,77 (13)
*6*	*Glukokortikoide als Dauertherapie (nicht, wenn sie bei Bedarf oder als Bridging-Therapie eingesetzt werden) ab einer Dosis von >* *7* *mg/Tag Prednisolon-Äquivalent erhöhen das CVD-Risiko bei RA und PsA und werden somit als „rot“ eingestuft* Die Evidenz zur CVD-Risikoerhöhung durch systemisch verabreichte Glukokortikoide ist robust. Ausdrücklich sei darauf hingewiesen, dass hier die Dauertherapie adressiert wird. Aus unserer Sicht spricht nichts gegen den Einsatz von Steroiden zum Bridging oder zur Behandlung von akuten Schüben. Obwohl in den Empfehlungen der Behandlung der Psoriasisarthritis vom Einsatz von Steroiden abgeraten wird, werden diese hier dennoch erwähnt, da es in der Praxis doch zu einem breiteren Einsatz dieser Medikamente kommen dürfte. Da die Evidenz für > 7 mg/Tag am besten sein dürfte, wurde dieser Cut-off gewählt, wobei es einzelne Studien gibt, die ein erhöhtes CVD-Risiko bereits bei geringeren Dosierungen zeigen [53–55]	9,5 (16)
*7*	*Methotrexat senkt das CVD-Risiko bei RA und wird somit als „dunkelgrün“ eingestuft* Methotrexat (MTX) stellt die Basistherapie der rheumatoiden Arthritis (RA) dar und ist nicht nur aufgrund seiner krankheitsmodifizierenden Effekte, sondern auch wegen möglicher kardioprotektiver Wirkung von uns als dunkelgrün eingestuft worden. Zahlreiche epidemiologische und klinische Studien weisen darauf hin, dass MTX mit einer signifikanten Reduktion des kardiovaskulären Risikos bei RA-Patient*innen assoziiert ist. Interessanterweise deuten einige Studien darauf hin, dass Methotrexat unabhängig von der Reduktion der Inflammation auch direkte kardioprotektive Effekte entfalten könnte. So wurden z. B. positive Effekte auf endotheliale Funktionen, oxidativen Stress und Lipidprofile beschrieben. Zusammenfassend kann unserer Recherche nach also nicht nur von einem kardiovaskulär sicheren, sondern auch potenziell kardioprotektiven Wirkstoff ausgegangen werden [56–59]	9,85 (13)
*8*	*Methotrexat senkt das CVD-Risiko bei PsA/perSpA und wird somit als „dunkelgrün“ eingestuft* Die Rolle von Methotrexat (MTX) zur Reduktion des kardiovaskulären Risikos bei PsA/perSpA ist weniger umfassend untersucht als bei RA, doch wächst die Evidenz, dass MTX auch bei PsA kardiovaskulär positive Effekte aufweist. Die aktuelle Datenlage zeigt, dass MTX-Therapie bei PsA-Patient*innen, mit einer signifikant niedrigeren Inzidenz kardiovaskulärer Ereignisse assoziiert ist – vor allem im Vergleich zu unbehandelten Patient*innen oder solchen unter NSAR-Monotherapie. Dabei werden sowohl indirekte Effekte über die entzündungshemmende Wirkung als auch mögliche direkte kardioprotektive Mechanismen diskutiert [56, 60–62]	9,85 (13)
*9*	*Sulfasalazin gilt als sicher bezüglich des CVD-Risikos in der Behandlung der RA und wird somit als „grün“ eingestuft* Hinsichtlich des kardiovaskulären Risikos gilt Sulfasalazin (SSZ) als neutral bis potenziell gering protektiv, wobei die Datenlage begrenzt ist. Es gibt keine Hinweise darauf, dass SSZ das kardiovaskuläre Risiko erhöht; in einigen Beobachtungsstudien wurde sogar eine geringe Risikoreduktion bei längerer Anwendung beschrieben. Der mögliche kardiovaskuläre Nutzen wird primär auf die entzündungshemmende Wirkung zurückgeführt, da eine Senkung der systemischen Inflammation allgemein als protektiv gegenüber atherosklerotischen Prozessen gilt. Allerdings fehlen klare Belege für direkte kardioprotektive Effekte von SSZ. Aufgrund dieser Evidenzlage wird Sulfasalazin im Rahmen der kardiovaskulären Risikobewertung als „grün“ eingestuft – also als sichere, aber nicht explizit schützende Therapieoption [63, 64]	9,92 (12)
*10*	*Sulfasalazin gilt als sicher bezüglich des CVD-Risikos in der Behandlung der PsA/perSpA und wird somit als „grün“ eingestuft* Sulfasalazin scheint analog zur rheumatoiden Arthritis auch in der Therapie der PsA und peripheren SpA sicher zu sein. Einige Studien bzw. In-vitro-Daten deuten sogar darauf hin, dass die Substanz mit einer signifikanten Reduktion des kardiovaskulären Gesamtereignisrisikos assoziiert ist. Diese potenziellen Vorteile sind auf die Kontrolle der systemischen Inflammation und auf eine Hemmung der Thrombozytenaggregation zurückzuführen. Aufgrund der Datenlage und der langjährigen klinischen Erfahrung wird SSZ in Bezug auf das CVD-Risiko somit als „grün“ eingestuft [61, 65]	9,92 (12)
*11*	*Leflunomid kann durch eine Steigerung des arteriellen Blutdrucks zu einem erhöhten CVD-Risiko in der Behandlung von RA führen und wird somit als „gelb“ eingestuft* Nachdem eine Leflunomid-Therapie in mehreren Studien mit einem Anstieg des arteriellen Blutdrucks assoziiert wurde – einem der wichtigsten modifizierbaren Risikofaktoren für kardiovaskuläre Ereignisse –, gilt es in Bezug auf das kardiovaskuläre Risiko nicht als gänzlich unbedenklich Beobachtungsstudien zeigten, dass unter LEF-Therapie ein signifikanter Blutdruckanstieg (> 10 mm Hg systolisch) auftreten kann. In einer großen Kohortenstudie war Leflunomid im Vergleich zu Methotrexat mit einem erhöhten Risiko für neu auftretende arterielle Hypertonie assoziiert Bezüglich harter kardiovaskulärer Endpunkte (Myokardinfarkt, Schlaganfall) ist die Datenlage hingegen weniger eindeutig. Dennoch bleibt der Blutdruckanstieg unter LEF ein gut dokumentierter und potenziell klinisch relevanter Risikofaktor Daher wird Leflunomid von uns als „gelb“ eingestuft – das heißt: Eine Anwendung ist nicht generell kontraindiziert, aber die engmaschige Überwachung des Blutdrucks und ggf. antihypertensive Begleittherapie sind essenziell, insbesondere bei vorbestehenden kardiovaskulären Risikofaktoren [66–68]	9,5 (12)
*12*	*Leflunomid kann durch eine Steigerung des arteriellen Blutdrucks zu einem erhöhten CVD-Risiko in der Behandlung von PsA (und perSpA) führen und wird somit als „gelb“ eingestuft* Ähnlich einer LEF-Therapie bei der RA wird diese auch bei der PsA mit einem Anstieg des arteriellen Blutdrucks in Verbindung gebracht In verschiedenen Kohorten und Registerstudien, teils populationsübergreifend (RA, PsA), wurde berichtet, dass bis zu 20 % der mit LEF behandelten Patient*innen eine neu auftretende oder sich verschlechternde arterielle Hypertonie entwickelten Obwohl konkrete prospektive Daten zur direkten Erhöhung harter kardiovaskulärer Endpunkte (z. B. Herzinfarkt, Schlaganfall) bei PsA unter Leflunomid fehlen, ist aufgrund der bekannten Hypertonie-induzierenden Wirkung ein erhöhtes CVD-Risiko nicht auszuschließen – insbesondere bei Patient*innen mit bereits bestehenden Risikofaktoren wie metabolischem Syndrom oder Diabetes mellitus, die in der PsA-Kohorte häufig vorkommen Daher wurde Leflunomid im Rahmen dieser Leitlinie hinsichtlich des kardiovaskulären Risikos bei PsA als „gelb“ eingestuft: Die Substanz ist nicht grundsätzlich kontraindiziert, erfordert jedoch eine regelmäßige Blutdruckkontrolle sowie ggf. die Einleitung oder Anpassung einer antihypertensiven Therapie. Bei Patient*innen mit instabiler Hypertonie oder manifester kardiovaskulärer Vorerkrankung sollte eine Nutzen-Risiko-Abwägung erfolgen und gegebenenfalls auf Alternativen ausgewichen werden [69]	9,67 (12)
*13*	*Hydroxychloroquin senkt das CVD-Risiko bei RA und wird somit als „dunkelgrün“ eingestuft* Hydroxychloroquin spielt zwar in der Therapie der RA nur mehr eine untergeordnete Rolle. Angesichts des so positiven Nebenwirkungsprofils wird trotzdem auf dieses Medikament eingegangen. In mehreren Studien konnte ein Effekt auf Surrogatparameter wie Cholesterin, Blutzucker und auch eine plättchenaggregationshemmende Wirkung nachgewiesen werden, wodurch sich eine CVD-Risiko-Reduktion ergibt [70–73]	10 (12)
*14*	*TNF-Inhibitoren senken das CVD-Risiko bei RA und werden somit als „dunkelgrün“ eingestuft. Zu beachten gilt, dass TNF-Inhibitoren bei Herzinsuffizienz (NYHA (New York Heart Association) III/IV) aufgrund möglicher Verschlechterung nicht eingesetzt werden sollen* Die beste Evidenz zur CVD-Risikoreduktion von TNF-Inhibitoren findet man in der Therapie der RA. Nicht nur Surrogatparameter, sondern offenbar auch die CVD-Ereignisrate scheint unter dieser Behandlung reduziert zu sein. Die Warnung zum Einsatz bei höhergradiger Herzinsuffizienz sollte allerdings beachtet werden. Neuere Studien zu diesem Risiko liegen unseres Wissens nicht vor [61, 74–76]	9,31 (13)
*15*	*TNF-Inhibitoren senken das CVD-Risiko bei PsA und werden somit als „dunkelgrün“ eingestuft. Zu beachten gilt, dass TNF-Inhibitoren bei Herzinsuffizienz (NYHA III/IV) aufgrund möglicher Verschlechterung nicht eingesetzt werden sollen* Die Evidenz zu TNF-Inhibitoren bei PsA ist verglichen mit jener zur RA geringer. Allerdings kann man auch hier von einer robusteren Datenlage ausgehen, weshalb auch hier eine positive Einschätzung zur CVD-Risikoreduktion abgegeben wurde [77–79]	9,31 (13)
*16*	*TNF-Inhibitoren senken das CVD-Risiko bei SpA und werden somit als „dunkelgrün“ eingestuft. Zu beachten gilt, dass TNF-Inhibitoren bei Herzinsuffizienz (NYHA III/IV) aufgrund möglicher Verschlechterung nicht eingesetzt werden sollen* Die Evidenz zur CVD-Risikoreduktion bei der SpA ist sehr gering und dem häufig noch jüngeren Alter und dem dadurch ohnehin noch geringen CVD-Risiko der SpA-Patient*innen geschuldet. Die Einschätzung zur CVD-Reduktion wurde teils auch aus der Therapie der RA und PsA extrapoliert [80–83]	9,31 (13)
*17*	*Abatacept gilt als sicher bezüglich des CVD-Risikos in der Behandlung der RA und wird somit als „grün“ eingestuft* Abatacept scheint ein günstiges CVD-Risikoprofil zu besitzen und in einzelnen Studien ähnlich zu TNF-Inhibitoren das CVD-Risiko sogar zu senken; die Evidenz hierfür ist allerdings nicht ausreichend, um Abatacept als besonders günstig einzustufen [84–86]	9,75 (12)
*18*	*Abatacept gilt als sicher bezüglich des CVD-Risikos in der Behandlung der PsA und wird somit als „grün“ eingestuft* Abatacept hat – obwohl zugelassen – in der Therapie der PsA eine eher untergeordnete Rolle. Dementsprechend lassen sich aufgrund fehlender Evidenz kaum Aussagen dazu treffen, ob das CVD-Risiko durch Abatacept sogar gesenkt werden könnte. Insofern speist sich dieses Statement aus den Sicherheitsdaten für diese Substanz [87]	9,75 (12)
*19*	*Rituximab gilt als sicher bezüglich des CVD-Risikos in der Behandlung der RA und wird somit als „grün“ eingestuft* Die Evidenz für eine möglicherweise besonders starke CVD-Risikosenkung durch Rituximab ist sehr gering. Rituximab scheint ein kardiovaskulär sicheres Präparat zu sein; wobei es einzelne Berichte über kardiovaskuläre Ereignisse kurz nach der Verabreichung dieser Therapie gibt – allerdings kann nicht differenziert werden, ob diese möglicherweise durch den Einsatz der üblicherweise eingesetzten Kortison-Prämedikation ausgelöst wurden [88, 89]	9,33 (12)
*20*	*Interleukin-6-Inhibitoren senken das CVD-Risiko bei RA und werden somit als „dunkelgrün“ eingestuft* Für IL(Interleukin)-6-Inhibitoren, insbesondere Tocilizumab, scheint ein sehr günstiges kardiovaskuläres Profil vorzuliegen. In mehreren Studien konnte eine Reduktion sowohl von kardiovaskulären Ereignisraten als auch von kardiovaskulären Surrogatparametern festgestellt werden. Die laut Literatur passagere Erhöhung der Cholesterinwerte scheint keinen nachteiligen Effekt auf das CVD-Risiko zu haben, vielmehr scheint sich das atherogene Profil der Lipidwerte unter Tocilizumab zu verbessern [90–93]	9,62 (13)
*21*	*Interleukin-1-Inhibitoren senken das CVD-Risiko bei RA und werden somit als „dunkelgrün“ eingestuft* Obwohl zugelassen, spielt die Interleukin-1-Hemmung in der Therapie der RA nur eine sehr untergeordnete Rolle. Deshalb liegen auch keine belastbaren Daten zum CVD-Risiko bei RA unter IL-1-Blockade (insbesondere Anakinra) vor. Die Einschätzung, dass IL-1-Blockade das CVD-Risiko senkt, stammt aus kardiologischen Studien, wo es durch Canakinumab zu einer Reduktion von CV-Ereignissen kam [94, 95]	9,77 (13)
*22*	*Interleukin-23- und Interleukin-12/23-Inhibitoren gelten als sicher bezüglich des CVD-Risikos in der Behandlung der PsA und werden somit als „grün“ eingestuft* Für IL-23-Inhibitoren gibt es zur CVD-Risikosenkung sehr wenig Evidenz; die vorhandenen Daten sind vor allem aus Safety-Studien übernommen. Allerdings gibt es zu Ustekinumab vereinzelte Fallberichte, die ein erhöhtes CVD-Risiko anzeigen; dies konnte allerdings in anderen Fallserien nicht bestätigt werden, wobei vereinzelt sogar eine Senkung des CVD-Risikos beschrieben wurde [96–99]	9,42 (12)
*23*	*Interleukin-17-Inhibitoren gelten als sicher bezüglich des CVD-Risikos in der Behandlung der PsA und werden somit als „grün“ eingestuft* Analog zu den IL-23-Inhibitoren gibt es auch hier nur sehr wenig Evidenz dafür, dass IL-17-Hemmer das CVD-Risiko besonders senken würden; auch hier wurde vor allem auf Basis der Sicherheitsdaten entschieden [100–102]	9,58 (12)
*24*	*Interleukin-17-Inhibitoren gelten als sicher bezüglich des CVD-Risikos in der Behandlung der SpA und werden somit als „grün“ eingestuft* Analog zu den IL-23-Inhibitoren gibt es auch hier nur sehr wenig Evidenz dafür, dass IL-17-Hemmer das CVD-Risiko besonders senken würden; auch hier wurde vor allem auf Basis der Sicherheitsdaten entschieden [103, 104]	9,58 (12)
*25*	*JAK(Januskinase)-Inhibitoren gelten als sicher bezüglich des CVD-Risikos in der Behandlung der RA und werden somit als „grün“ eingestuft, sollten aber laut derzeitigen Leitlinien nur nach vorangegangener Prüfung des CVD-Risikos und von Alternativen eingesetzt werden* Die Evidenz zur kardiovaskulären Sicherheit von JAK-Inhibitoren ist aufgrund der von der ORAL SURVEILLANCE-Studie verursachten Diskussion insgesamt sehr gut. Die durch die genannte Studie suspizierte Erhöhung des CVD-Risikos von Tofacitinib (verglichen mit Adalimumab) bei Patient*innen über 65 Jahren konnte in keiner anderen Studie repliziert werden. Vielmehr zeigt sich in Real-Life- und Register-Daten sowie in den Sicherheitsdaten zu den anderen in der Rheumatologie eingesetzten JAK-Inhibitoren (Baricitinib, Filgotinib, Upadacitinib) kein Hinweis auf eine Erhöhung des Risikos. Die Oral Surveillance-Studie führte dazu, dass ein Warnhinweis in den aktuellen Leitlinien ausgesprochen wurde, weshalb dieser auch in diesem Konsensus mitaufgenommen wurde. Allerdings gibt es bereits konkrete Stellungnahmen zur JAK-Therapie der DGRH (Deutsche Gesellschaft für Rheumatologie und klinische Immunologie) und auch einen aktuellen europäischen Konsensus, der diesem – nicht replizierten – CVD-Risiko mehr oder weniger widerspricht. Ein weiterer Aspekt in der Therapie mit JAK-Inhibitoren aus kardiovaskulärer Sicht ist eine geringe Erhöhung der Lipidwerte nach Beginn der Therapie. Allerdings konnte bis dato kein Hinweis auf eine dadurch erhöhte CVD-Ereignisrate gesehen werden [105–109]	9,08 (12)
*26*	*JAK-Inhibitoren gelten als sicher bezüglich des CVD-Risikos in der Behandlung der PsA und SpA und werden somit als „grün“ eingestuft, sollten aber laut derzeitigen Leitlinien nur nach vorangegangener Prüfung des CVD-Risikos und von Alternativen eingesetzt werden* Die Datenlage zur CVD-Sicherheit und CVD-Risikoreduktion ist verglichen mit jener zur RA bei JAK-Inhibitoren weit geringer. Insofern muss von den Safety-Daten und auch von jenen bei der RA extrapoliert werden. Jedenfalls gibt es kein Anzeichen für ein erhöhtes CVD-Risiko unter JAK-Inhibitoren bei PsA oder SpA [10, 110]	9,17 (12)
*27*	*Apremilast gilt als sicher bezüglich des CVD-Risikos in der Behandlung der PsA und wird somit als „grün“ eingestuft* Apremilast scheint ein metabolisch sehr günstiges Nebenwirkungsprofil wie z. B. Gewichtsabnahme zu besitzen. Anhand der Sicherheitsdaten konnte kein relevantes kardiovaskuläres Risiko gezeigt werden; allerdings findet sich bis dato auch kein Hinweis auf eine substanzielle Senkung von kardiovaskulären Endpunkten [111–114]	9,67 (12)
*28*	Bei allen Patient*innen *sollte zunächst ein etwaiges Lipidparadox ausgeschlossen werden* Vor Beginn eines CVD-Screenings (sollte nur in Remission oder bei niedriger Krankheitsaktivität durchgeführt werden) sollte durch ein Messen der Krankheitsaktivität ein Lipidparadox ausgeschlossen werden. Bei rheumatischen Erkrankungen mit hoher Aktivität kann ein sehr niedriges Gesamt- und LDL-Cholesterin vorliegen, wodurch ein niedriges CVD-Risiko vorgetäuscht werden kann. Im Sinne des Lipidparadoxes ist aber gerade bei diesen Patient*innen das CVD-Risiko deutlich erhöht. Durch die Therapie der Grunderkrankung können die Cholesterinwerte wieder ansteigen, was aber nicht unbedingt eine Erhöhung des CVD-Risikos mit sich bringt. Vielmehrt ist eine Risikosenkung durch eine Besserung des atherogenen Index zu erwarten [12, 13]	9 (16)
*29*	*Die LDL-steigernde Wirkung einzelner Medikamente sollte beachtet werden* Vor allem für IL-6-Inhibitoren und auch für JAK-Inhibitoren wurde eine Steigerung des LDL-Cholesterins beschrieben. Diese findet vor allem in den ersten 6 Monaten nach Therapiebeginn statt und scheint auch reversibel zu sein. Vor allem für Tocilizumab wurde nachgewiesen, dass es dadurch zu keiner erhöhten Ereignisrate kommt. Darüber hinaus scheint sich der atherogene Index zu verbessern [16–19]	9,69 (16)
*30*	*Polypharmazie und Arzneimittelinteraktionen sollen vermieden werden* Polypharmazie (= die gleichzeitige Einnahme von 5 oder mehr Medikamenten) kann einerseits die Compliance und Therapieadhärenz negativ beeinflussen, andererseits kann es auch zu relevanten Arzneimittelnebenwirkungen kommen. Das Vermeiden von Polypharmazie, auch durch den Einsatz von Kombinationspräparaten, kann zu einer Verbesserung der Adhärenz führen [14, 15]	9,94 (16)
*31*	*Es sollte regelmäßig eine kardiale Anamnese (Dyspnoe, AP-Symptomatik, Palpitationen, Synkopen etc.) erhoben werden* Ziel dieses Konsensus ist eine Detektion von CVD-Erkrankungen bei asymptomatischen Patient*innen mit rheumatischen Erkrankungen. Patient*innen mit Symptomen einer CVD-Erkrankung sollten primär bei entsprechenden Spezialist*innen in Abklärung stehen. Da an Rheuma erkrankte Patient*innen in regelmäßigem Kontakt mit Rheumatolog*innen stehen, empfehlen wir trotzdem in regelmäßigen Abständen das Durchführen einer kardialen Anamnese, um nicht berichtete Symptome oder nicht diagnostizierte Ereignisse anamnestisch zu erheben. Das Intervall, in welchen Abständen diese durchgeführt werden soll, ist unserer Ansicht nach individuell zu entscheiden. Ein Abfragen solcher Symptome bei jeder Visite ist aus unserer Sicht bei asymptomatischen Patient*innen allerdings nicht zielführend [20]	9,21 (14)
*32*	Alle Patient*innen sollten eine Beratung zu Lifestyle-Modifikation (Nikotinstopp, Gewichtsabnahme, körperliche Aktivität etc.) erhalten Um – analog zur Abgabe von Lebensstilempfehlungen in Phase 1 – die Wichtigkeit eines gesunden Lebensstiles zu unterstreichen, sollte die Bedeutung von CV-Risikofaktoren, wie z. B. Nikotinabusus oder Übergewicht, in der Phase des CVD-Screenings noch einmal wiederholt werden [115]	9,94 (16)
*33*	Alle Patient*innen ab dem 40. Lebensjahr sollen einem CVD-Screening unterzogen werden Der Zeitpunkt, ab wann ein CVD-Screening bei Rheuma-Patient*innen erstmals durchgeführt werden soll, ist nicht eindeutig definiert. Da die offiziellen CVD-Risikorechner der ESC ab dem 40. Lebensjahr anzuwenden sind, wurde dieses Alter als Cut-off gewählt. Bei Patient*innen, bei denen kardiovaskuläre Hochrisikosituationen vorliegen, kann individuell auch ein früherer Zeitpunkt gewählt werden [21]	9,88 (16)
*34*	*Das CVD-Screening soll in einem Intervall von 3 bis 5 Jahren wiederholt werden (bei grundlegenden Veränderungen früher)* Dieses Statement orientiert sich an den derzeit gültigen Empfehlungen der EULAR. In diesen wird auch empfohlen, dass ein Screening bei grundlegenden Veränderungen auch öfter als alle 3 bis 5 Jahre durchgeführt werden kann [6, 11]	9,5 (16)
*35*	Bei allen Patient*innen soll einmalig Lipoprotein(a) bestimmt werden Aufgrund der zunehmenden Bedeutung einer einmaligen Messung des Lipoprotein(a) in der CVD-Risikobewertung wurde dieser Wert in den Workflow für Rheumapatient*innen mit aufgenommen. Weiters soll ein erhöhtes Lp(a) (> 30 mg/dl oder > 75 nmol/l) als Upgrade-Kriterium dienen, wodurch – nach erfolgtem Screening durch den SCORE2(-OP) – eine Einstufung in eine höhere Risikogruppe erfolgen kann [28, 116, 117]	9,69 (16)
*36*	*Gescreent wird anhand des SCORE2/SCORE2-OP, um das 10-Jahres-Risiko eines CV-Ereignisses bzw. CV-assoziierten Todes zu erheben* Da ein für alle Rheumapatient*innen und Erkrankungen einheitliches und validiertes CVD-Screening-Tool noch fehlt, wird auf das offizielle CVD-Tool der ESC referenziert. Dies kann ab dem 40. Lebensjahr eingesetzt werden (bzw. SCORE2-OP ab dem 70. Lebensjahr) [22, 23]	9,63 (16)
*37*	*Das jeweilige Ergebnis soll bei der rheumatoiden Arthritis mit dem Faktor 1,5 multipliziert werden* Um eine genauere Risikoabschätzung bei RA-Patient*innen zu ermöglichen, wird dem durch die RA erhöhten CVD-Risiko insofern Rechnung getragen, indem der durch den SCORE2(-OP) errechnete Wert mit dem Faktor 1,5 multipliziert wird. Dies wurde auch in den Empfehlungen der EULAR und der ESC festgehalten. Da die Evidenz für eine Differenzierung des RA-Risikos zwischen seropositiver und seronegativer RA gering ist, wird darauf auch in diesem Konsensus vorerst verzichtet [1, 6, 9]	9,5 (16)
*38*	*Das jeweilige Ergebnis soll bei der Psoriasisarthritis mit dem Faktor 1,5 multipliziert werden* Das erhöhte CVD-Risiko durch eine Psoriasisarthritis ist gut belegt. Auch aufgrund der gleichzeitig oft assoziierten metabolischen Störungen wie Dyslipidämie, Adipositas oder Hyperurikämie scheint eine Multiplikation des SCORE2-Ergebnisses mit dem Faktor 1,5 gerechtfertigt [1, 9]	9,06 (16)
*39*	*Das jeweilige Ergebnis soll bei radiographischer axialer SpA mit dem Faktor 1,5 multipliziert werden – in der Annahme, dass radiographische Veränderungen Ausdruck einer über längere Zeit insuffizient behandelten Grunderkrankung sind und daher das CVD-Risiko erhöht ist* Bei Patient*innen mit radiographischer axialer Spondyloarthritis (raxSpA) ist davon auszugehen, dass die sichtbaren strukturellen Veränderungen häufig eine längere Phase unzureichend kontrollierter Krankheitsaktivität widerspiegeln. Chronische systemische Inflammation stellt einen etablierten Risikofaktor für kardiovaskuläre Erkrankungen dar. Daher wird empfohlen, das durch SCORE2 bzw. SCORE2-OP berechnete 10-Jahres-Risiko für kardiovaskuläre Ereignisse mit dem Faktor 1,5 zu multiplizieren. Diese Empfehlung erfolgt in Analogie zur Vorgehensweise bei RA und PsA und wurde in Zusammenarbeit mit dem Arbeitskreis Spondyloarthritis entwickelt [1, 9, 10]	9,62 (13)
*40*	*Anhand des SCORE2/SCORE2-OP-Screenings werden die Patientinnen unterschiedlichen Risikogruppen zugeordnet mit konsekutiver Festlegung von entsprechenden LDL-Zielwerten. Bei niedrigem CV-Risiko soll ein LDL-Zielwert von <* *116* *mg/dl angestrebt werden. Bei moderatem CV-Risiko soll ein LDL-Zielwert von <* *100* *mg/dl angestrebt werden. Bei hohem CV-Risiko soll ein LDL-Zielwert von <* *70* *mg/dl angestrebt werden. Bei sehr hohem CV-Risiko soll ein LDL-Zielwert von <* *55* *mg/dl angestrebt werden* Die Einteilung der Patient*innen in kardiovaskuläre Risikogruppen erfolgt auf Basis des SCORE2- bzw. SCORE2-OP-Algorithmus. Abhängig von der Risikokategorie sollen evidenzbasierte LDL-Zielwerte zur Primärprävention verfolgt werden, wie sie auch von der ESC empfohlen werden. Diese Zielwerte betragen: < 116 mg/dl bei niedrigem, < 100 mg/dl bei moderatem, < 70 mg/dl bei hohem und < 55 mg/dl bei sehr hohem kardiovaskulärem Risiko. Eine konsequente Umsetzung dieser Zielwerte soll zur Reduktion zukünftiger kardiovaskulärer Ereignisse beitragen und wird somit auch im rheumatologischen Kontext als essenziell angesehen [24]	9,75 (16)
*41*	*Ergänzend sollen Upgrade-Kriterien definiert und in die Risikostratifizierung miteinbezogen werden. Sollten mehr als eine Autoimmunerkrankung gleichzeitig vorliegen oder ein erhöhtes Lipoprotein(a) (Grenzwerte >* *30* *mg/dl oder >* *75* nmol/l) oder eine andere Erkrankung, die ein hohes kardiovaskuläres Risiko mit sich bringt, kann der/die Patient*in eine Risikogruppe höher als das eigentliche SCORE2-Ergebnis eingestuft werden Zusätzlich zur rechnerischen Risikoabschätzung mittels SCORE2 bzw. SCORE2-OP sollen individuelle Risikofaktoren berücksichtigt werden, um eine adäquate Stratifizierung zu gewährleisten. Als Upgrade-Kriterien gelten insbesondere das Vorliegen multipler Autoimmunerkrankungen, pathologisch erhöhte Lipoprotein(a)-Werte sowie weitere relevante Komorbiditäten mit bekannter kardiovaskulärer Risikosteigerung. In diesen Fällen erscheint eine Einstufung in die nächsthöhere Risikogruppe gerechtfertigt, um der tatsächlichen kardiovaskulären Gefährdung besser Rechnung zu tragen und entsprechend strengere Therapieziele zu definieren [1, 26–28]	9,8 (15)
*42*	*Bei Grunderkrankungen mit einem vordefinierten LDL-Ziel (wie z.* *B. bei familiärer Hypercholesterinämie oder bei Diabetes mellitus) soll die rheumatologische Erkrankung als Upgrade-Kriterium dienen* Liegt eine Grunderkrankung mit bereits definierter kardiovaskulärer Hochrisikosituation vor – wie beispielsweise bei familiärer Hypercholesterinämie oder bei Diabetes mellitus – soll eine zusätzlich bestehende entzündlich rheumatische Erkrankung als weiteres Upgrade-Kriterium anerkannt werden. Diese Konstellation rechtfertigt eine Einstufung in eine höhere Risikokategorie mit entsprechend strengeren LDL-Zielwerten, um der additiven Wirkung beider Erkrankungen auf das kardiovaskuläre Risiko angemessen zu begegnen [24]	9,92 (13)
*43*	*Bei Auffälligkeiten in der kardialen Anamnese wird eine weiterführende Diagnostik bei dem/der entsprechenden Spezialisten/-in empfohlen* Rheumapatient*innen sollten bei Auffälligkeiten in der kardialen Anamnese von Kardiolog*innen untersucht und betreut werden. Die CV-Verantwortlichkeit der Rheumatolog*innen endet in diesem Fall, da eine spezialisierte kardiologische Betreuung erforderlich ist. Eine enge interdisziplinäre Zusammenarbeit ist hierbei essenziell, um die bestmögliche Versorgung der Patienten zu gewährleisten [118, 119]	9,69 (15)
*44*	*Je nachdem, in welche Risikogruppe der/die Patient/in eingeordnet wird, sollte eine dem individuellen Risiko angepasste weiterführende kardiovaskuläre Diagnostik eingeleitet werden* Das Ziel dieses Konsensus soll nicht nur eine primärprophylaktische Therapie sein, sondern auch eine gezielte, dem jeweiligen CVD-Risiko entsprechende Diagnostik	9,56 (16)
*45*	Bei Auffälligkeiten in der weiterführenden Diagnostik sollte eine Zuweisung zu FÄ (Fachärzt*innen) für Kardiologie/in eine kardiologische Ambulanz erfolgen Bei pathologischen Befunden in der risikobasierten weiterführenden Diagnostik sollte fachspezifische Expertise (Kardiologie, Angiologie etc.) eingeholt und die entsprechenden Maßnahmen sollten von den zuständigen Expert*innen durchgeführt werden. Bei Auffälligkeiten oder Erkrankungen aus anderen Fachgebieten sollte diesbezüglich die Führung der Patient*innen abgegeben oder interdisziplinär geteilt werden [119]	9,94 (16)
*46*	*Bei niedrigem Risiko sollten regelmäßige Blutdruckmessungen empfohlen werden* Bei niedrigem CV-Risiko sollte aufgrund der hohen Prävalenz einer arteriellen Hypertonie in der Allgemeinbevölkerung und unter Patient*innen mit rheumatischen Erkrankungen in regelmäßigen Abständen der arterielle Blutdruck gemessen werden. Das Blutdruckziel sollte sich an den Zielen für die Allgemeinbevölkerung orientieren, da für Patient*innen, die an einer RA/PsA/SpA erkrankt sind, keine speziellen Empfehlungen vorliegen [11, 29, 30]	9,25 (16)
*47*	*Bei moderatem CVD-Risiko sollten zusätzlich zu Blutdruckmessungen auch eine Karotissonographie, bei hohem und sehr hohem Risiko zusätzlich ein EKG durchgeführt werden* Bei moderatem CVD-Risiko sollte zusätzlich zu Blutdruckmessungen auch eine Karotissonographie durchgeführt werden, um atherosklerotische Veränderungen an den Karotiden frühzeitig erkennen zu können. Aufgrund der hohen Verfügbarkeit dieser Untersuchung wird die Karotissonographie schon bei moderatem Risiko empfohlen. Im Falle von Plaquebildungen sollte sich die weiterführende Therapie (LDL-Ziel) an den derzeitigen Leitlinien (LDL-Ziel < 55 mg/dl) orientieren [5, 31] Ein routinemäßiges EKG-Screening bei allen asymptomatischen Patient*innen sämtlicher CV-Risikogruppen würde in Relation zu einer geringen Anzahl pathologischer, aber zu einer hohen Zahl unspezifischer Befunde führen, die wiederum unnötige Folgeuntersuchungen nach sich ziehen könnten. Deshalb haben wir uns im Sinne eines gezielten Ressourceneinsatzes und einer Vermeidung von Überdiagnostik für den Einsatz eines EKGs erst im Stadium des hohen und sehr hohen Risikos entschieden [35–38]	9,8 (16)
*48*	*Um Vorhofflimmern oder andere Rhythmusstörungen zu detektieren, sollte ein opportunistisches Screening mittels Palpation des Pulses, EKG und gegebenenfalls elektronischer Devices erfolgen. Um eine Herzinsuffizienz zu erkennen, sollten eine liberale Messung des NT-proBNP und bei erhöhten Werten eine Echokardiographie erfolgen* Die Prävalenz klinisch bedeutsamer kardialer Manifestationen abseits der Atherosklerose, wie z. B. Vorhofflimmern, Perikarditis, AV-Blockierungen oder Herzinsuffizienz, ist bei Rheumapatient*innen ebenso erhöht Wir empfehlen gemäß den ESC-Empfehlungen ein opportunistisches Screening mittels Palpation des Pulses; ein Ruhe-EKG kann nichtinvasive Hinweise auf Vorhofflimmern oder andere Rhythmusstörungen liefern. In weiterer Folge sollten elektronische Devices (Langzeit-EKG etc.) eingesetzt werden Um eine Störung der Pumpfunktion bzw. eine kardiale Insuffizienz frühzeitig zu erkennen, sollte eine liberale Bestimmung des NT-proBNP erfolgen. Bei Auffälligkeiten sollte zu einer Echokardiographie zugewiesen werden Die transthorakale Echokardiographie liefert essenzielle Informationen über Klappenfunktionen, Perikarditis, die systolische und diastolische Funktion des linken und rechten Ventrikels oder auch eine pulmonale Hypertension. Sie ist insbesondere bei rheumatischen Erkrankungen mit bekanntem Risiko für eine Herzbeteiligung von hoher Relevanz. So können z. B. eine Aortenklappeninsuffizienz bei ankylosierender Spondylitis oder eine Perikarditis bei RA frühzeitig erkannt werden [32, 34]	10 (16)
*49*	*Bei hohem kardiovaskulärem Risiko und weiterhin bestehender niedriger Krankheitsaktivität sollte ein noch konsequenteres Streben nach einer Remission stattfinden* Daten belegen, dass das CVD-Risiko mit dem Grad der Krankheitsaktivität assoziiert ist. Bei hohem oder sehr hohem CVD-Risiko und gleichzeitig noch bestehender, wenn auch niedriger Krankheitsaktivität sollte dieser Risikofaktor durch eine – wenn möglich – noch intensivere antirheumatische Behandlung minimiert werden [120–123]	9,63 (16)
*50*	Primär sollten Statine, Ezetimib und auch Bempedoinsäure durch Rheumatolog*innen verordnet werden. Bei Nichterreichen des LDL-Ziels sollte die Zuweisung zu Lipidspezialist*innen erfolgen Die Einleitung einer Lipid-senkenden Therapie kann (aber muss nicht) bereits über FÄ für Rheumatologie erfolgen. Orientierend an den lokalen Gegebenheiten (Erstattung, Bewilligung etc.) sollten in Österreich primär Statine, Ezetimib und Bempedoinsäure als orale Medikamente verordnet werden. Falls ein LDL-Ziel mit diesen Mitteln nicht erreicht werden sollte und andere Medikamente (z. B. PCSK-9-Inhibitoren) notwendig sein sollten, ist eine Zuweisung zu Lipidspezialist*innen anzuraten (Lipidambulanzen etc.) [24, 115]	9,19 (16)

*LoA* Level of Agreement

## Ergebnisse in Fragen zusammengefasst

### Welche Patient*innen sollen kardiovaskulär untersucht und betreut werden?

An sich sollte das kardiovaskuläre Risiko bei allen Patient*innen mit rheumatischen Erkrankungen beachtet werden. In diesem Konsensus haben wir uns auf die rheumatologisch relevantesten bzw. häufigsten Erkrankungen (RA, PsA, SpA) konzentriert. Zu beachten ist, dass das Screening primär asymptomatische Patient*innen adressiert. Das heißt, Patient*innen mit Beschwerden oder bekannten kardiovaskulären Erkrankungen sollten in fachärztlicher (kardiologischer) Betreuung stehen.

### Wie soll die Behandlung des kardiovaskulären Risikos in den klinischen Alltag integriert werden?

**Abb. 4:**
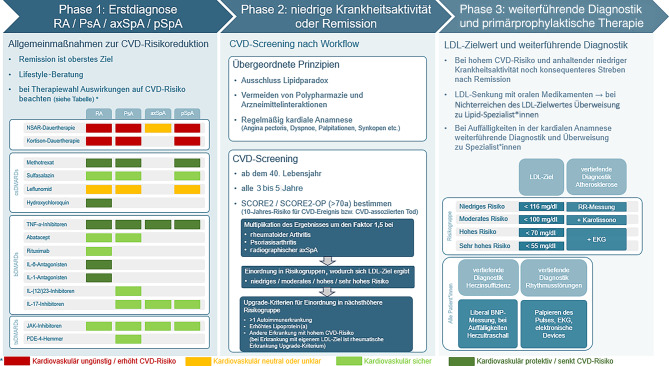
Zusammenfassung des Workflows

Unserer Meinung nach sollte das Management des CVD-Risikos schrittweise an die einzelnen Phasen der rheumatischen Erkrankungen angepasst werden (Abb. 4).

In einer *ersten Phase*, in der die Krankheitsaktivität der RA/PsA/SpA noch im Vordergrund steht, sollte aus kardiovaskulärer Sicht vor allem die Wahl eines diesbezüglich günstigen Medikamentes in Erwägung gezogen werden (Abb. 1).

In einer *zweiten Phase*, in der die Patient*innen in einem Status niedriger Krankheitsaktivität oder in Remission sind, sollte ein strukturiertes CV-Screening durchgeführt werden. Hierfür wurden übergeordnete Prinzipien formuliert, und es wird die Verwendung des SCORE2(-OP) der ESC empfohlen. Das Ergebnis des Screenings ergibt das 10-Jahres-Risiko für CV-Erkrankungen und CV-assoziierten Tod. Da bei Rheumapatient*innen ein höheres Risiko als bei der Normalbevölkerung vorliegt, wird dieses Ergebnis mit einem Multiplikationsfaktor versehen. Bei RA, PsA und bei radiographischer SpA beträgt dieser 1,5. Dadurch ergibt sich das individuelle CV-Risiko (niedrig, moderat, hoch oder sehr hoch) (Abb. 2).

In einer *dritten Phase erfolgt* je nach Einordnung in die jeweilige Risikogruppe eine Empfehlung für ein konkretes LDL-Cholesterin-Ziel und auch eine Empfehlung für eine dem Risiko angepasste weiterführende Diagnostik (Abb. 3)

### Wie ist der Einfluss der antirheumatischen Therapie auf das CV-Risiko?

In mehreren Untersuchungen konnte nachgewiesen werden, dass eine erhöhte KH-Aktivität eng mit einem erhöhten CV-Risiko assoziiert ist. Umgekehrt wird durch das Erreichen einer niedrigen Krankheitsaktivität oder Remission auch das CV-Risiko gesenkt.

Die Evidenz für den Einfluss der antirheumatischen Medikation auf das CVD-Risiko ist sehr unterschiedlich. So gibt es für die RA aus CVD-Sicht eine relativ gute Datenlage. Die (sehr oft deckungsgleiche) Therapie wurde aber bei der PsA und auch bei der SpA weit weniger häufig diesbezüglich untersucht. So musste im vorliegenden Konsensus oft aus den Daten der RA für die PsA und SpA rückgeschlossen werden. Für einzelne Medikamente wurden Daten aus den Safety-Studien herangezogen, da keine spezifischen Untersuchungen zur Auswirkung auf das CV-Risiko vorliegen.

### Wie und wann erfolgt das CVD-Screening?

Im vorliegenden Konsensus wird ein CVD-Screening aus pragmatischen Gründen ab dem 40. Lebensjahr empfohlen, da die Datenlage zur präventiven LDL-senkenden Therapie vor dem 40. Lebensjahr unzureichend ist und auch der SCORE2 erst ab dem 40. Lebensjahr Anwendung findet. Das Screening sollte alle 3 bis 5 Jahre wiederholt werden. Wichtig ist zu betonen, dass es sich um ein Screening handelt, das heißt, dass hier nur kardiovaskulär asymptomatische Patient*innen eingeschlossen werden. Patient*innen mit Symptomen oder kardiovaskulären Vorerkrankungen sollten spezifisch durch Fachärzt*innen (z. B. Kardiolog*innen) betreut werden. In speziellen Fällen kann es Sinn machen, dass das CV-Screening bereits früher oder auch häufiger durchgeführt wird.

Wir empfehlen das Screening-Tool der ESC (SCORE2 bzw. SCORE2-OP), um eine einheitliche und pragmatische Verwendung zu gewährleisten. Es gibt zwar für einzelne rheumatische Erkrankungen entwickelte Screening-Tools, die sich aber bis jetzt in der Praxis nicht durchgesetzt haben. Der Vorteil des SCORE2(-OP) ist, dass er (bis auf den Blutdruck und das Cholesterin) gänzlich ohne Voruntersuchungen gemacht werden kann.

Das Ergebnis (= 10-Jahres-Risiko für CVD oder CV-assoziierten Tod) soll mit einem der speziellen rheumatologischen Grunderkrankung entsprechenden Faktor multipliziert werden (1,5 für RA, 1,5 für PsA und 1,5 für raxSpA).

### Was sind die Konsequenzen des CVD-Screenings?

In unserem Konsensus ergeben sich aus der Einordnung in eine Risikogruppe im Wesentlichen 2 Konsequenzen. Zum einen wird ein der Risikogruppe entsprechendes Ziel für das individuelle LDL-Cholesterin formuliert. Andererseits erfolgt auch eine individuelle Empfehlung für eine weiterführende kardiovaskuläre Diagnostik. Bei niedrigem CVD-Risiko sollte der Blutdruck regelmäßig gemessen werden; bei moderatem Risiko sollte zusätzlich zur Blutdruckmessung eine Karotissonographie erfolgen. Bei hohem und sehr hohem CVD-Risiko sollte zusätzlich zu den schon genannten Untersuchungen ein EKG veranlasst werden. Um frühzeitig eine Herzinsuffizienz erkennen zu können, sollte liberal NT-proBNP bestimmt werden, bei erhöhten Werten sollte zu einer Echokardiographie zugewiesen werden. Um Rhythmusstörungen, wie z. B. Vorhofflimmern, zu erkennen, sollten der Puls palpiert, ein EKG durchgeführt oder elektronische Devices angewendet werden.

### Was passiert bei einem auffälligen Ergebnis in der Diagnostik bzw. bei Beschwerden?

Im Falle von kardiovaskulär anmutenden Beschwerden in der Anamnese sowie pathologischen Ergebnissen in der weiterführenden Diagnostik (Blutdruckmessungen, Karotissonographie, NT-pro-BNP, EKG, Echokardiographie) sollte umgehend eine Zuweisung zu spezialisierten Fachärzt*innen oder in jeweilige Ambulanzen erfolgen. Die weitere Diagnostik im Rahmen einer kardiologischen Begutachtung orientiert sich an den jeweiligen kardiologischen Leitlinien (z. B. Echokardiographie bei erhöhtem BNP etc.).

Die primäre Zuständigkeit von Rheumatolog*innen endet an diesem Punkt.

### Gibt es Therapieziele zu traditionellen CVD-Risikofaktoren wie Cholesterin, Blutdruck, Lebensstil?

Es erscheint uns wichtig zu betonen, dass eine Lebensstilberatung für Patient*innen mit rheumatischen Erkrankungen und kardiovaskulärem Risiko von großer Bedeutung ist und darauf absolut nicht verzichtet werden sollte. Ein gesunder Lebensstil kann potenziell die Krankheitsaktivität reduzieren und hat Effekte auf die kardiovaskuläre Gesundheit und die Lebensqualität. Deshalb erfolgt eine Betonung dieser Maßnahmen sowohl in Phase 1 als auch in Phase 2 unseres Workflows.

Da es bezüglich des arteriellen Blutdruckes kein individualisiertes Ziel für diese 3 rheumatischen Erkrankungen gibt, empfehlen wir schon bei niedrigem Risiko zumindest eine regelmäßige Kontrolle der Blutdruckwerte und eine Einstellung auf den Zielbereich, der für die Normalbevölkerung definiert wurde.

Die Therapieziele der Dyslipidämie und somit vor allem das LDL-Ziel ergeben sich aus der Einordnung in die jeweiligen Risikogruppen. Auch Rheumatolog*innen können bereits (aber müssen nicht) eine Cholesterin-senkende Therapie beginnen; unserer Meinung nach sollte sich aber hier die Therapie auf den Einsatz oral verfügbarer Medikamente wie vor allem Statine, Ezetimib oder auch Bempedoinsäure beschränken. Bei therapierefraktären Fällen sollten Spezialist*innen hinzugezogen werden. Falls es für Rheumatolog*innen nicht möglich ist, selbst die Cholesterin-senkende Therapie einzuleiten, ist jedenfalls die Formulierung des individuellen LDL-Ziels auf Basis des Screening-Ergebnisses essenziell, um eine gezielte interdisziplinäre Betreuung zu gewährleisten.

Die ESC formulierte in ihren Empfehlungen auch ein Screening auf Diabetes mellitus im Sinne einer Bestimmung von Blutzucker oder HbA_1c_. Falls noch kein Diabetes mellitus bekannt sein sollte und eine länger dauernde Therapie mit systemischen Glukokortikoiden verwendet werden sollte, soll auf jeden Fall auch bei Rheumapatient*innen ein Screening auf einen (steroidinduzierten) Diabetes mellitus in Betracht gezogen werden.

## Diskussion

Der vorliegende Konsensus stellt den Versuch dar, eine individualisierte und pragmatische Handlungsanleitung für den klinischen Alltag bereitzustellen (Abb. 4). Ausgehend von den derzeitigen EULAR-Empfehlungen, sind viele verschiedene Strategien zur CVD-Risikoreduktion denkbar.

Schon die Befolgung der Leitlinien für die Allgemeinbevölkerung würde eine Verbesserung der Situation bringen, da derzeit sicher von einer Unterversorgung auszugehen ist. Dies würde jedoch das durch rheumatische Erkrankungen veränderte Risiko nicht abbilden.

Auf der anderen Seite des Spektrums wäre im Extremfall die Einschätzung des CVD-Risikos je nach Erkrankung, Dauer der Erkrankung und Summe der Aktivität über diesen Zeitraum als möglicherweise genaueste Risikoeinschätzung denkbar. Dies würde allerdings jeglichen zeitlichen Rahmen sprengen. Insofern haben wir versucht, eine im Alltag durchführbare Strategie zu entwickeln. Diese soll zu konkreten Empfehlungen zum LDL-Ziel und zu weiterführenden kardiovaskulären Screening-Untersuchungen führen, die an das individuelle Risiko angepasst sind.

Die *Schwächen *dieses pragmatischen Zugangs sind evident. Eines der Grundprobleme ist die nicht eindeutig geklärte konkrete Erhöhung des CVD-Risikos dieser 3 Erkrankungen, wofür die Heterogenität der Studienlage verantwortlich ist. So wurden häufig unterschiedliche Outcome-Parameter definiert (oft auch keine harten Endpunkte, sondern Surrogatparameter). In einzelnen Publikationen wurden nicht nur atherosklerotische Manifestationen, sondern auch z. B. kardiale Insuffizienz oder Arrhythmien unter dem CVD-Outcome subsumiert. Oft fehlen auch Daten zum Phänotyp und zur Aktivität der Grunderkrankung (seropositiv/-negativ? Aktivität?); des Weiteren stammen manche Daten aus der Präbiologikaära und sind heute möglicherweise so nicht mehr reproduzierbar.

Die *Evidenzlage zur kardiovaskulären Risikoreduktion der antirheumatischen Medikamente* ist ebenfalls teilweise sehr niedrig. Die meisten Daten sind aus der Therapie der rheumatoiden Arthritis generiert worden. Bei der Psoriasisarthritis und vor allem der Spondyloarthritis finden sich nur wenige bis gar keine Studien, die Aussage zur CVD-Reduktion einzelner Therapien geben können. So musste vereinzelt die Einordnung auch anhand der Safety-Daten aus den Zulassungsstudien und Registerdaten sowie durch die Risikoreduktion, die sich durch eine effektive Behandlung der Grunderkrankung ergibt, geschlussfolgert beziehungsweise eingeschätzt werden. Weiters stellt sich die Frage, ob Hinweise für etwaige Nebenwirkungen einzelner Medikamente wirklich robust aufgearbeitet wurden (z. B. Leflunomid und Blutdruckerhöhung), und welche Rolle einzelne Basismedikamente in der Realität tatsächlich spielen (Hydroxychloroquin bei RA, Abatacept bei PsA, Anakinra bei RA etc.); die diesbezügliche Wertigkeit wurde im vorliegenden Konsensus bewusst nicht hinterfragt. Ebenso finden sich – zumindest unseres Wissens – in der Literatur keine Untersuchungen zu antirheumatischen Kombinationstherapien hinsichtlich der Auswirkung auf das kardiovaskuläre Risiko. Genauso wenig wurde auch die Kombination von DMARDs (disease modifying antirheumatic drug) mit Statinen (denen pleiotrope Effekte zugeschrieben werden) genau aufgearbeitet.

Weitere Schwächen sind in Phase 2 des Workflows zu finden. So ist die Empfehlung eines CVD-Screenings ab dem 40. Lebensjahr als ein pragmatischer Zugang zu verstehen, ebenso die Empfehlung, dies alle 3 bis 5 Jahre zu wiederholen. Für einzelne Patient*innen in Hochrisikokonstellationen kann es Sinn machen, ein Screening früher oder umfangreicher durchzuführen. Ein weiterer Kritikpunkt sind sicherlich die vorgeschlagenen *Multiplikationsfaktoren *für das Ergebnis des SCORE-2(-OP)-Ergebnisses. Diese Empfehlungen ergeben sich einerseits aus den EULAR- und ESC-Empfehlungen für die RA; für die PsA wurden diese aufgrund der Datenlage zum CVD-Risiko, aber auch aufgrund der Assoziation mit metabolischen Störungen übernommen. Für die Spondyloarthritis ist die Lage hierfür viel weniger klar, weshalb in einem pragmatischen Zugang der Faktor 1,5 für Patient*innen mit radiographischer axSpA empfohlen wird. Nach eingehender Literatursuche ist die Annahme argumentierbar, dass bei radiographischer axSpA eine lange Zeit unzureichend behandelte SpA mit einer hohen inflammatorischen Last aktiv war oder ist, und sich daraus ein erhöhtes CVD-Risiko ergibt. Dieser Punkt wurde gemeinsam mit dem Spondyloarthritis-Arbeitskreis diskutiert.

Welche Komorbiditäten als *Upgrade-Kriterien* verwendet werden, wurde ebenso bewusst offengelassen, da eine genaue Auflistung von Erkrankungen mit erhöhtem CVD-Risiko außerhalb der Rheumatologie den Rahmen dieses Konsensus sprengen würde. Ebenso ist eine genaue Definition des Lipoprotein(a)-Grenzwertes möglicherweise durch zukünftige Anpassungen der lipidologischen Empfehlungen zu hinterfragen.

In Summe fehlt eine breite Evidenz für eine positive Auswirkung auf CV-assoziierte Erkrankungen durch eine strengere Lipideinstellung von Rheuma-Patient*innen. Ein Hinweis für eine Wirksamkeit lässt sich aus einer Subgruppenanalyse der Oral Surveillance-Studie ableiten. Hier zeigten jene Patient*innen, die zusätzlich zu Tofacitinib auch Statine erhielten, ein in etwa gleich großes CVD-Risiko wie jene, die nur Adalimumab erhielten; jedenfalls zeigte sich eine Verbesserung zu den Patient*innen, die nur Tofacitinib (und kein Statin) in der Therapie aufwiesen. In einer Analyse des Fourier-Trials konnte gezeigt werden, dass eine LDL-Senkung durch den PCSK9-Inhibitor Evolocumab zu einer stärkeren CVD-Risikoreduktion bei Patient*innen mit Autoimmunerkrankungen verglichen mit Patient*innen ohne Autoimmunerkrankungen führt [124].

Sicherlich finden sich in Phase 3 Kritikpunkte bezüglich der Wertigkeit oder Sinnhaftigkeit der weiterführenden Diagnostik. In diesem pragmatischen Zugang sollen lediglich Untersuchungstechniken empfohlen werden, die von Rheumatolog*innen leicht durchführbar beziehungsweise durch Zuweisungen leicht zugänglich sind. Tiefergehende, spezialisiertere Diagnostik soll den jeweiligen Fachrichtungen (Kardiologie, Angiologie, Radiologie etc.) vorbehalten sein. Das Blutdruckmonitoring bei geringem Risiko stellt jedenfalls einen Grundpfeiler in der Risikoreduktion dar und ist in der allgemeinen Inneren Medizin gut etabliert. Unstrittig ist die Karotissonographie ein Surrogatparameter für eine allgemeine Atherosklerose (wie auch eine koronare Herzerkrankung) und bildet auch das Risiko für zerebrale Ischämien ab. Die Wertigkeit des EKG zur Detektion einer KHK (koronare Herzerkrankung) ist bei asymptomatischen Patient*innen laut Literatur zu hinterfragen. Deshalb wurde das EKG nur bei Patient*innen mit hohem bis sehr hohem Risiko angeführt; eine zusätzliche Indikation besteht zum Screening für Rhythmusstörungen (wofür auch das Palpieren des Pulses und das Verwenden kardiologischer elektronischer Devices, wie z. B. auch ein Langzeit-EKG, empfohlen sind).

Die liberale Messung des NT-pro-BNP soll unserer Meinung nach zur Frühdiagnostik einer kardialen Insuffizienz dienen, die in der Literatur ebenso gehäuft vorzukommen scheint. In weiterer Folge bildet die Echokardiographie das Herzinsuffizienzrisiko bei Rheumatiker*innen (wie auch das Risiko für Perikarditis sowie pulmonalarterielle Hypertension) und das erhöhte Aortenklappeninsuffizienzrisiko bei SpA-Patient*innen ab. Darüber hinaus hat die Echokardiographie auch einen gewissen Wert zur Erkennung eines Vorhofflimmerns (durch den PW-Doppler über der Mitralklappe und auch die Größe des linken Vorhofs).

Falls Patient*innen über Beschwerden im Sinne von z. B. Angina pectoris berichten sollten, ist natürlich umgehend eine kardiale Diagnostik und hier selbstredend sehr rasch der Einsatz eines EKGs angezeigt.

Ein potenzieller Kritikpunkt ist die Aussage, dass bei Patient*innen, die ein hohes bis sehr hohes CVD-Risiko haben und weiterhin eine zumindest niedrige Krankheitsaktivität der rheumatischen Grunderkrankung aufweisen, ein noch intensiveres Streben nach Remission stattfinden soll. Dies soll unserer Meinung nach den früheren Einsatz von (kardial besonders günstigen) bDMARDs (biologische DMARDs) nahelegen.

## Zusammenfassung

Der vorliegende Konsensus stellt eine individualisierte, pragmatische und praxisnahe Handlungsanleitung dar, um das CVD(„cardiovascular disease“)-Risiko bei Patient*innen mit rheumatoider Arthritis (RA), Psoriasisarthritis (PsA) oder Spondyloarthritis (SpA) zu reduzieren. Zusammenfassend scheint es uns wichtig zu betonen, dass die kardiovaskuläre Betreuung dieser Patient*innen ein kontinuierlicher Prozess ist, weshalb dieser von uns in drei Phasen gegliedert wurde. Das Erreichen einer anhaltenden Remission scheint jedenfalls eine der wichtigsten kardioprotektiven Maßnahmen zu sein.

Zukünftig ist eine Verbesserung der Evidenzlage wünschenswert, möglicherweise durch eine Vereinheitlichung von Studienendpunkten.

Um Empfehlungen zur Risikoreduktion bei allen rheumatischen Erkrankungen verfassen zu können, sollte jedoch Klarheit über den Pathomechanismus von Myokardischämien im Rahmen der jeweiligen unterschiedlichen Erkrankungen geschaffen werden.

Eine Zukunftsvision wäre sicherlich eine gemeinsame Strategie unterschiedlicher Fachgesellschaften, die von einem erhöhten CVD-Risiko ihrer Patient*innen betroffen sind, wie zum Beispiel die Gastroenterologie, Dermatologie oder auch die Nephrologie.
